# Supplementary material for: mtDNA heteroplasmy level and copy number indicate disease burden in m.3243A>G mitochondrial disease
Source: EMBO Mol Med. 2018 May 7;10(6):e8262. doi: 10.15252/emmm.201708262 (PMC5991564; doi:10.15252/emmm.201708262)
Supplement: Supplementary file 1 — Appendix [file EMMM-10-e8262-s001.pdf]

Appendix

Contents **Page**

Supplementary Method 1: Development of a method to adjust urine m.3243A>G heteroplasmy levels for sex	2
Figure S1: Production of sex-adjusted urine heteroplasmy levels.	3
Supplementary Method 2: Development of a method to adjust blood m.3243A>G heteroplasmy levels for age	4
Table S1: Comparisons of the association of different heteroplasmy measures with total disease burden (A) and progression (B).	5
Table S2: Demographics of the study population	6
Reference	7

Supplementary Method 1:

Development of a method to adjust urine m.3243A>G heteroplasmy levels for sex

Given the clear sex effect in urine m.3243A>G heteroplasmy levels, we reasoned that sex-adjusted urine heteroplasmy levels would be of more benefit than unadjusted levels and, therefore, devised a method for producing sex-adjusted levels using data from subjects in whom both urine and muscle heteroplasmy levels had been measured (N = 75).

1. The logit of the mean raw urine heteroplasmy levels were taken to ensure that adjusted heteroplasmy levels remain within a realistic range (i.e. 0-100%; **Figure S1A**).
2. Linear regression was performed to estimate relationship between muscle and urine heteroplasmy. To ensure that a 1% change in muscle heteroplasmy is equivalent to a 1% change in urine, logit urine levels were then divided by the gradient (0·791; **Figure S1B**).
3. Linear regression was performed to determine the sex-specific intercepts. Note that the gradient is 1, due to step 2 (**Figure S1C**). To adjust for the higher levels in males, logit urine levels in females were adjusted by subtracting the female intercept (-0·608) and males levels by subtracting the male intercept (0·625).
4. The logit function was then reversed, producing percentage heteroplasmy levels (**Figure S1D**). The gradient is now normalised, so that a 50% muscle heteroplasmy is equivalent to a 50% adjusted urine heteroplasmy. Sex-specific intercepts are also normalised, so adjusted urine heteroplasmy and muscle heteroplasmy are nominally equivalent in both sexes, i.e. adjusted urine levels represent a surrogate for muscle heteroplasmy levels.

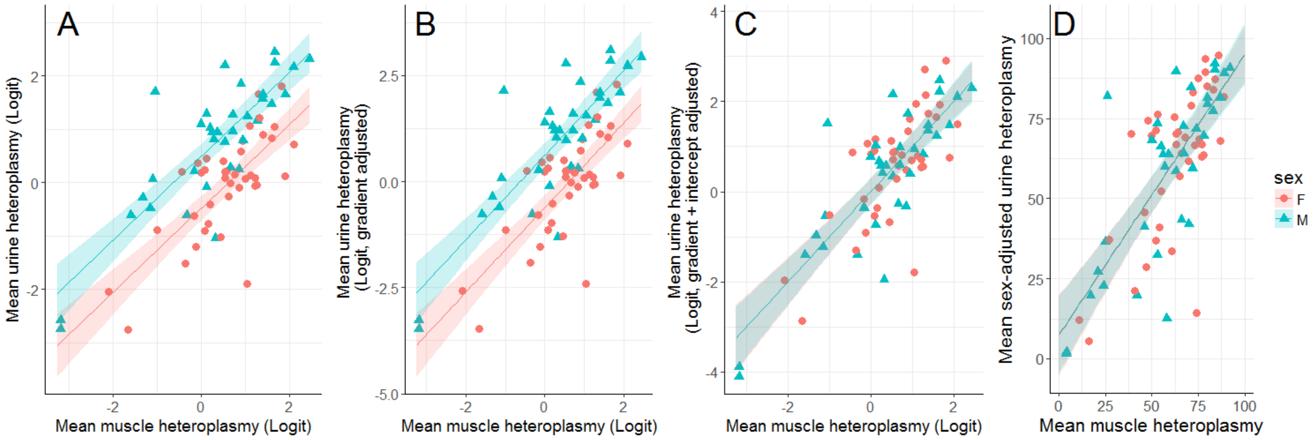

Figure S1: Production of sex-adjusted urine heteroplasmy levels. All graphs show separate regression lines and 95% confidence intervals for males and females. **(A)** The logit of mean urine heteroplasmy regressed against the logit of mean muscle heteroplasmy and sex (slope = 0·791, 95% CI = 0·648, 0·934, P<0·001). **(B)** Regression after the gradient was adjusted to ensure equality between logit muscle and urine heteroplasmy. **(C)** Regression after intercepts adjusted for sex-specific effects. **(D)** Regression of adjusted levels after converting back to percentage heteroplasmy level.

Supplementary Method 2:

Development of a method to adjust blood m.3243A>G heteroplasmy levels for age

Rajisimha *et al.* proposed the following correction:[1]

$het_{adjusted} = het_{blood}*exp(S*age_{blood})$, where S = 0·02.

$1/e^{s}$ (0·9802) is an exponential decline factor (edf), describing a yearly decline in blood heteroplasmy levels of around 2%. Using sex-adjusted urine and blood heteroplasmy levels (N=204; individuals over 16 years of age with heteroplasmies of at least 10% in urine and 2% in blood) we can calculate an exponential decline factor for each individual using this formula.

$edf = exp(ln(het_{blood}/het_{urine})/(age_{blood}))$

To represent all data, the edf must be independent of both age and urine heteroplasmy level. Linear regression showed the edf to be independent of het_{urine} (P = 0·96) but not age_{blood} (P<0·001). Therefore, we employed the following formula:

$edf = exp(ln(het_{blood}/het_{urine})/(age_{blood} + x)^{y})$

where x is an age adjuster that increases the number of years to account for a higher initial rate of decline and y represents compound decline rate adjuster, which essentially slows the rate of decline over time. We tested different values of x (range 0-18) and y (range 0·65-1).

The two models that minimised the relationship of the edf with age and heteroplasmy were:

$edf = exp(ln(het_{blood}/het_{urine})/(age_{blood} + 12))$, median = 0·977, 95% CI = 0·976, 0·979

and

$edf = exp(ln(het_{blood}/het_{urine})/(age_{blood})^{0·75})$, median = 0·927, 95% CI = 0·923, 0·932

The first represents a compound decline of around 2% a year but accounts for a rapid reduction at the beginning by adding 12 years. The second represents a compound decline of around 7% a year at age zero, with a reduction in decline rate each year. These two proposed models are on a continuum; there are infinite models between that also minimise the relationship, however, in the interests of simplicity and as the first model is similar to the decline we observe in blood, we chose this model.

Therefore, we propose the following formula for age-correction of blood heteroplasmy levels:

$het_{adj} = het_{age}/0·977^{(age+12)}$

Table S1: Comparisons of the association of different heteroplasmy measures with total disease burden (A) and progression (B).

N	Heteroplasmy used in model		R^2_{first} - R^2_{second}	95% CI	P value
**A: Total disease burden**					
	**First**	**Second**			
210	Blood	Urine	0·093	0·016, 0·174	0·007
210	Blood	Age-adjusted blood	0.020	-0.020, 0.063	0.173
210	Age-adjusted blood	Sex-adjusted urine	0·055	-0·005, 0·117	0·036
210	Age-adjusted blood	Urine	0·073	0·011, 0·142	0·009
210	Sex-adjusted urine	Urine	0·018	-0·019, 0·058	0·166
210	Age-adjusted blood	Mean adjusted blood and urine	0.008	-0.018, 0.036	0.277
69	Muscle	Age-adjusted blood	0·022	-0·053, 0·095	0·304
69	Muscle	Blood	0·048	-0·026, 0·126	0·124
69	Muscle	Sex-adjusted urine	0·002	-0·088, 0·077	0·467
69	Muscle	Urine	0·006	-0·083, 0·089	0·460
69	Muscle	Mean adjusted blood and urine	0.007	-0.075, 0.078	0.416
**B: Disease progression**					
**N**	**Heteroplasmy used in model**		**Mean AIC_{first} - AIC_{second}**	**95% CI**	**P value**
	**First**	**Second**			
210	Age-adjusted blood	Sex-adjusted urine	-7·66	-16·78, 0·85	0·037
210	Age-adjusted blood	Urine	-9·99	-18·88, -1·36	0·010
210	Sex-adjusted urine	Urine	-2·67	-8·00, 2·17	0·181
210	Mean adjusted blood and urine	Age-adjusted blood	-0.64	-4.94, 3.40	0.624
69	Muscle	Age-adjusted blood	-2·05	-6·84, 2·60	0·189
69	Muscle	Sex-adjusted urine	-1·04	-5·71, 3·37	0·297
69	Muscle	Urine	-1·23	-6·58, 3·93	0·320
69	Muscle	Mean adjusted blood and urine	-1.08	-5.15, 3.06	0.696

Table S2: Demographics of the study population

	Number subjects
**Sex**	
Male	95
Female	147
**NMDAS**	
<5	30
≥5	195
Incomplete	17
**Age**	
18.0-25.0	26
25.1-35.0	50
35.1-45.0	52
45.1-55.0	59
55.1-65.0	42
65.1-75.0	11
75.1-85.0	2

Reference

1. Rajasimha HK, Chinnery PF, Samuels DC. Selection against pathogenic mtDNA mutations in a stem cell population leads to the loss of the 3243A-->G mutation in blood. *American journal of human genetics* 2008; **82**(2): 333-43.
